# Supplementary figures and images for: Molecular epidemiology of SARS-CoV-2 in Cyprus
Source: PLoS One. 2021 Jul 21;16(7):e0248792. doi: 10.1371/journal.pone.0248792 (PMC8294526; doi:10.1371/journal.pone.0248792)

**S1 Fig. Case-fatality rate (CFR) in Cyprus from March 2020 until January 2021**

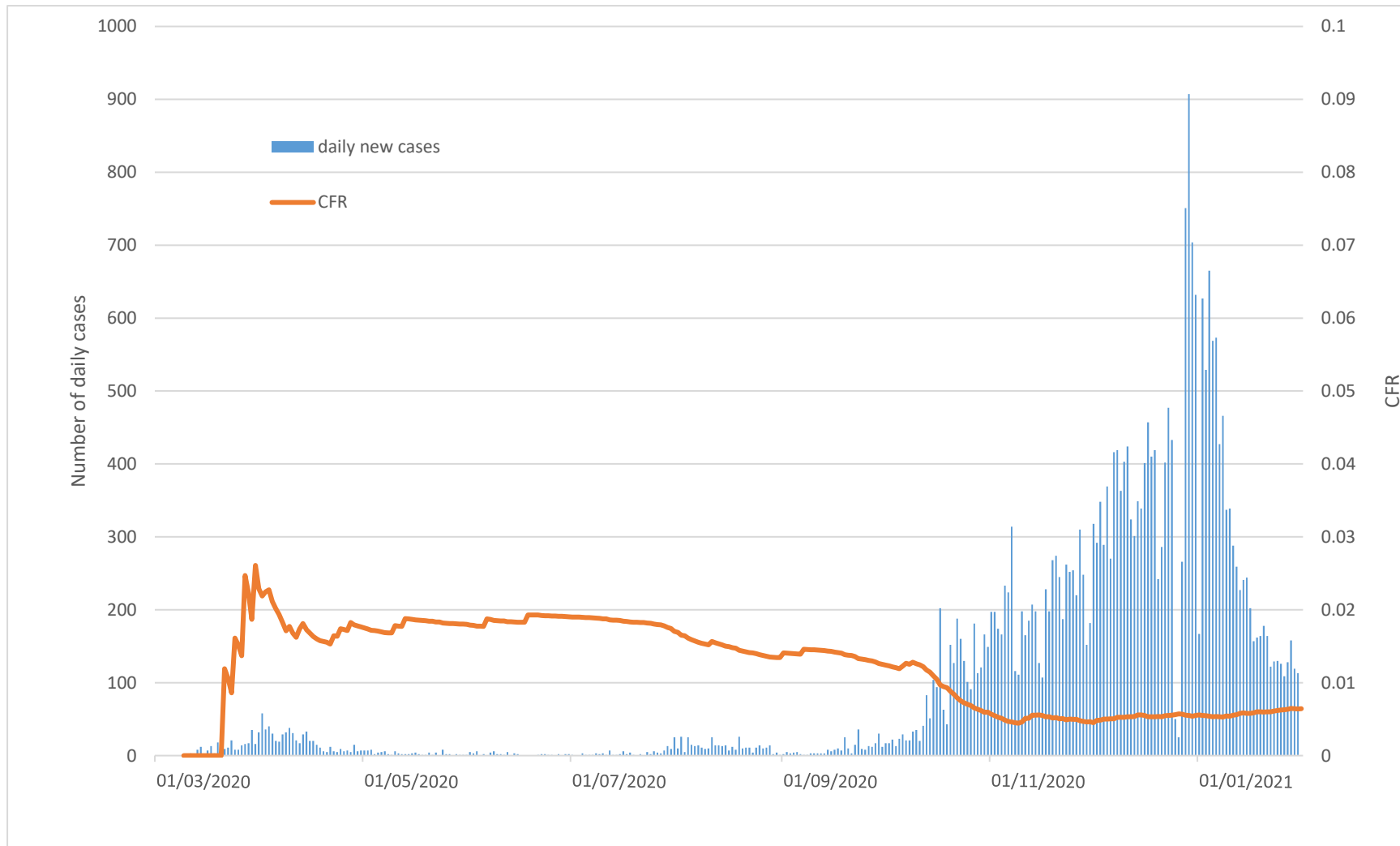

Supplement: S1 Fig — The CFR was calculated as the ratio between cumulative number of confirmed deaths and cumulative confirmed cases. The daily number of cases are shown for comparison. (PDF) [file pone.0248792.s001.pdf]
