# Supplementary material for: Molecular epidemiology of SARS-CoV-2 in Cyprus
Source: PLoS One. 2021 Jul 21;16(7):e0248792. doi: 10.1371/journal.pone.0248792 (PMC8294526; doi:10.1371/journal.pone.0248792)
Supplement: S1 Table — Single nucleotide polymorphisms (SNPs) were identified in the genomes with respect to the reference sequence (NC_045512). The frequency of SNPs is calculated by the number of sequences with the SNP/total number of sequences per lineage. SNPs are ordered by their position on the genome. Number of sequences of each lineage are indicated in brackets in the header. SNPs with frequency >75% are marked in green, SNPs with frequency between 50% and 75% are marked in yellow. (PDF) [file pone.0248792.s002.pdf]

S1 Table. Frequency of SNPs in lineages observed

Region	SNP	B.1 (1)	B.1.1.103 (2)	B.1.1.192 (1)	B.1.1.251 (2)	B.1.1.277 (3)	B.1.1.288 (1)	B.1.1.67 (1)	B.1.1.7 (10)	B.1.1.74 (5)	B.1.177 (9)	B.1.2 (17)	B.1.235 (1)	B.1.236 (7)	B.1.258 (72)	B.1.36 (1)
5'UTR	C66T	0	0	0	0	0	0	0	0	0	0.11	0	0	0	0	0
	C106T	0	0	0	0	0	0	0	0	0	0	0.94	0	0	0	0
	A187G	0	0	0	0	0	0	0	0	0	0	0	1	0	0	0
	C203T	0	0	1	0	0	0	0	0	0	0	0	0	0	0	1
	G204T	0	0	0	0	0	0	0	0	0	0.89	0	0	0	0	0
	C241T	1	1	1	1	1	1	1	1	1	1	1	1	1	1	1
	G434A	0	1	0	0	0	0	0	0	0	0	0	0	0	0	0
	T445C	0	0	0	0	0	0	0	0	0	1	0	0	0	0	0
	G529T	0	0	0	0	0	0	0	0	0	0.11	0	0	0	0	0
	A594G	0	0	0	0	0	0	0	0	0	0	0	0	0	0	1
	T595C	0	0	0	0	0	0	1	0	0	0	0	0	0	0	0
	C673T	0	0	1	0	0	0	0	0	0	0	0	0	0	0	0
	T694A	0	0	0	0	0	0	0	0	0	0	0	0	0	0.01	0
	G748T	0	0	0	0	0	0	0	0.1	0	0	0	0	0	0	0
	C829T	0	0	0	0	0	0	0	0	0	0	0	0	0	0	0
	C913T	0	0	0	0	0	0	0	1	0	0	0	0	0	0	0
	C1059T	0	0	0	0	0	0	0	0	0	0	1	0	0	0	0
	C1102T	0	0	0	0	0	0	0	0	0	0.11	0	0	0	0	0
	G1157A	0	0	0	0	0	0	0	0	0	0	0	0	0	0.07	0
	A1163T	0	1	0	0	0	0	0	0	0	0	0	0	0	0	0
	C1191T	0	0	0	0	0	0	0	0	0	0	0	0	0	0.01	0
	C1201T	0	0	0	0	0	0	0	0	0	0	0	1	0	0	0
	G1268A	0	0	0	0	0	0	0	0	0	0	0	0	0	0.04	0
	C1346T	0	0	0	0	0	0	0	0	0	0	0	0	0	0.03	0
	C1457T	0	0	0	0	0	0	0	0	0	0	0	0	0	0.01	0
	C1498T	0	0	0	0	0	0	0	0	0	0	0	0	0	0.04	0
	T1576C	0	0	0	0	0	0	0	0	0	0	0	0	0	0.04	0
	C1601T	0	0	1	0	0	0	0	0	0	0	0	0	0	0	0
	T1927C	0	0	0	0	0	0	0	0	0	0	0.94	0	0	0	0
	A1987G	0	0	0	0	0	0	0	0	0	0.11	0	0	0	0	0
	G1999C	0	0	0	0	0	0	0	0	0	0	0	0	0	0	1
	C2062T	0	0	0	0	0	0	0	0	0	0	0	0	0	0.01	0
	G2155T	0	0	0	0	0	0	0	0	0	0.11	0	0	0	0	0
	G2159C	0	0	0	0	0	0	0	0	0	0	0	0	0	0.01	0
	A2406G	0	0	0	0	0	0	0	0	0	0	0	0	0	0.01	0
	C2453T	0	0	0	0	0	0	0	0.9	0	0	0	0	0	0	0
	C2509T	0	0	0	0	0	0	0	0	0	0.11	0	0	0	0	0
	G2612A	0	0	0	0	0	0	0	0	0	0	0	0	0	0.03	0
	C2623Y	1	0	0	0	0	0	0	0	0	0	0	0	0	0	0
	G2625T	0	0	0	0	0	0	0	0	0	0	0	0	0	0.01	0
	A2869G	0	0	0	0	1	0	0	0	0	0	0	0	0	0	0
	C3037T	1	1	1	1	1	1	1	1	1	1	1	1	1	1	1
	C3045Y	0	0	0	0	0	0	0	0	0	0	0.06	0	0	0	0
	T3067C	0	0	0	0	0	0	0	0	0	0.22	0	0	0	0	0
	C3267T	0	0	0	0	0	0	0	1	0	0	0	0	0	0	0
	C3330T	0	0	1	0	0	0	0	0	0	0	0	0	0	0	0
	C3411T	0	0	0	1	0	0	0	0	0	0	0	0	0	0	0
	A3450G	0	0	0	0	0	0	0	0	0	0	0	0	0	0.01	0
	C3505T	0	0	0	0	0	0	0	0	0	0	0	0	0	0.04	0
	C3743T	0	0	0	0	0	0	0	0	0	0	0	0	0	0.17	0
	C3987T	0	0	1	0	0	0	0	0	0	0	0	0	0	0	0
	G4136T	0	0	0	0	0	0	0	0.9	0	0	0	0	0	0	0
	C4234T	0	0	0	0	0	0	0	0	0	0	0	0	0	0.01	0
	C4331T	0	0	0	0	0	0	0	0	0	0	0	0	0	0.01	0
	C4543T	0	0	0	0	0	0	0	0	0	0	0	0	0	0.01	0
	C4596T	0	0	0	0	0	0	0	0	0	0	0	1	0	0	0

S1 Table. Frequency of SNPs in lineages observed

	C4810T	0	0	0	0	0	0	0	0	0	0.11	0	0	0	0	0
	C4876T	0	0	0	0	0	0	0	0	0	0	0	0	0.14	0	0
	C4927T	0	0	0	0	0	1	0	0	0	0	0	0	0	0	0
	A4981G	0	0	0	0	0	0	0	0	0	0	0	0	0	0.03	0
	T5077C	0	0	0	0	0	0	0	0.9	0	0	0	0	0	0	0
	G5108A	0	0	0	0	0	0	0	0	0.2	0	0	0	0	0	0
	C5388A	0	0	0	0	0	0	0	1	0	0	0	0	0	0	0
	A5431G	0	0	0	0	0	0	0	0	0	0	0	0	0	0.01	0
	C5672T	0	0	0	0	0	0	0	0	0	0	0	0	0	0.01	0
	C5784T	0	0	0	0	0	0	0	0	0	0	0	0	0	0.01	0
	C5835T	0	0	0	0	0	0	0	0	0	0	0	0	1	0	0
	G5950T	0	1	0	0	0	0	0	0	0	0	0	0	0	0	0
ORF1a	C5986T	0	0	0	0	0	0	0	1	0	0	0	0	0	0	0
	G5990A	0	0	0	0	0	0	0	0	0	0	0	0	0	0.03	0
	G6052A	0	0	0	0	0	0	0	0	0	0	0.06	0	0	0	0
	C6286T	0	0	0	0	0	0	0	0	0	1	0	0	0	0	0
	C6317T	0	0	0	0	0	0	0	0	0	0.11	0	0	0	0	0
	T6353C	0	0	0	0	0	0	0	0	0	0	0	0	0	0.03	0
	A6418G	0	0	0	0	0	0	0	0	0	0	0	0	0	0.01	0
	C6636T	0	0	0	0	0	0	0	0	0.2	0	0	0	0	0	0
	T6954C	0	0	0	0	0	0	0	1	0	0	0	0	0	0	0
	C7081A	0	0	0	0	0	0	0	0	0	0	0	0	0	0.01	0
	C7173T	0	0	0	0	0	0	0	0	0	0	0	0	0	0.01	0
	T7767C	1	0	0	0	0	0	0	0.1	0	0	0	0	0	1	0
	C7834T	0	0	0	0	0	0	0	0	0	0	0	0	0	0.01	0
	C7926T	0	0	0	0	0	0	0	0	0	0.22	0	0	0	0	0
	C8047T	1	0	0	0	0	0	0	0	0	0	0	0	0	1	0
	C8092T	0	0	0	0	0	0	0	0	0	0	0	0	0	0.01	0
	C8208T	0	0	1	0	0	0	0	0	0	0	0	0	0	0	0
	A8244G	0	0	0	0	0	0	1	0	0	0	0	0	0	0	0
	G8408A	0	0	0	0	0	0	1	0	0	0	0	0	0	0.03	0
	C8481T	0	0	0	0	0	0	0	0	0	0	0	0	0	0.54	0
	A8848C	0	0	0	0	0	0	0	0	0	0	0	0	0	0.01	0
	T9271C	0	0	0	0	0	0	0	0	0	0	0	0	0	0.01	0
	A9509G	0	0	0	0	0	0	0	0	0	0	0	0	0	0.01	0
	C9532T	0	0	0	0	0	0	0	0	0	0	0	0	0	0.01	0
	C9661T	0	0	0	0	0	0	0	0	0	0	0	0	0	0.01	0
	C9693T	0	0	0	0	0	1	0	0	0	0	0	0	0	0	0
	C9745T	0	0	0	0	0	0	0	0	0	0.22	0	0	0	0	0
	A10225G	0	0	0	0	0	0	0	0	0	0	0	0	0	0.03	0
	C10319T	1	0	0	0	0	0	0	0	0	0	1	0	0	0	0
	A10323G	0	0	0	0	0	0	0	0	0	0.11	0	0	0	0.01	0
	C10543T	0	0	0	0	0	0	0	0	0	0	0	0	0	0.03	0
	G10610A	0	0	0	0	0	0	0	0	0	0	0	0	0	0.01	0
	C10626T	0	0	0	0	0	0	0	0	0	0	0	0	0	0.01	0
	C10641T	0	0	0	0	0	0	0	0	0	0	0	0	0	0.03	0
	C10833T	0	0	0	0.5	0	0	0	0	0	0	0	0	0	0	0
	G10870T	0	0	0	0	0	0	0	0	0	0.11	0	0	0	0.01	0
	T10906C	0	0	0	0	0	0	0	0	0	0	0.06	0	0	0	0
	G11083T	0	0	0	0	0	0	0	0	0	0	0	0	0	0.04	0
	C11173T	0	0	0	0	0	0	0	0	0	0	0	0	0	0.01	0
	G11335T	0	0	0	0	0	0	0	0	0	0	0.18	0	0	0	0
	G11417T	0	0	0	0	0	0	0	0	0	0	0	0	0	0.01	0
	C11530T	0	0	0	0	0	0	0	0	0	0	0	0	0	0.01	0
	A11533G	0	0	0	0	0	0	0	0	0	0.11	0	0	0	0	0
	G11557T	0	0	0	0	0	0	0	0	0	0	0	0	0	0.94	0
	T11587C	0	0	0	0	0	0	0	0	0	0	0	0	0	0.01	0
	C11747T	0	0	0	0	0	0	0	0	0	0	0	0	0	0.01	0
	G11761T	0	0	0	0	0	0	0	0	0	0	0	0	0	0.01	0
	G11771A	0	0	0	0	0	0	0	0	0	0	0	0	0	0.01	0
	A11789G	0	0	0	0	0	0	0	0	0.2	0	0	0	0	0	0

S1 Table. Frequency of SNPs in lineages observed

	C11824T	0	0	0	0	0	0	0	0	0	0	0	0	0	0.01	0
	G11842A	0	0	0	0	0	0	0	0	0	0	0	0	1	0	0
	A12048G	0	0	0	0	0	0	0	0	0	0	0	0	0	0.01	0
	A12159G	0	0	0	0	0	0	0	0.6	0	0	0	0	0	0	0
	G12191T	0	0	0	0	0	0	1	0	0	0	0	0	0	0	0
	C12400T	0	0	0	0	0	0	0	0	0	0	0	0	0	0.04	0
	T12447C	0	0	0	0	0	0	0	0	0	0	0	0	0	0.01	0
	C12525T	0	0	0	0	0	0	0	0.1	0	0	0	0	0	0	0
	C12663T	0	0	0	0	0	0	0	0	0	0	0	0	0	0.01	0
	A12759G	0	0	0	0	0	0	0	0	0	0	0	0	1	0	0
	G12988T	1	0	0	0	0	0	0	0	0	0	0	0	0	1	0
	C13168Y	0	0	0	0	0	0	0	0	0	0	0	0	0	0.01	0
	C13342T	0	0	0	0	0	0	0	0	0	0.22	0	0	0	0	0
	C13458Y	0	0	0	0	0	0	0	0	0	0	0	0	0	0.01	0
	C13620T	0	0	0	0	0	0	0	0	0	0	0	0	0	0.01	0
	A13768G	0	0	1	0	0	0	0	0	0	0	0	0	0	0	0
	T13779C	0	0	0	0	0	0	0	0	0	0	0.94	0	0	0	0
	C13945A	0	0	0	0	0	0	0	0	0	0.11	0	0	0	0	0
	A14012G	0	0	0	0	0	0	0	0	0	0	0	0	0	0.01	0
	G14055T	0	0	0	0	0	0	0	0	0	0.22	0	0	0	0	0
	A14213G	0	0	0	0	0	0	0	0	0	0	0	0	0	0.01	0
	A14240C	0	0	0	0	0	0	0	0	0	0	0	0	0	0.01	0
	C14408T	1	1	1	1	1	1	1	1	1	1	1	1	1	1	1
	C14676T	0	0	0	0	0	0	0	1	0	0	0	0	0	0	0
	C14697T	0	0	0	0	0	0	0	0	0	0	0	0	0	0	0
	C14889T	0	0.5	0	0	0	0	0	0	0	0	0	0	0	0	0
	C14925T	0	0	0	0	0	0	0	0	0	0	0	0	0	0.01	0
	T15096C	0	0	0	0	0	0	0	0.1	0	0	0	0	0	0	0
	G15181A	0	0	0	0	0	0	0	0	0	0	0.12	0	0	0	0
	C15279T	0	0	0	0	0	0	0	1	0	0	0	0	0	0	0
	A15301T	0	0	0	0	0	0	0	0	0.2	0	0	0	0	0	0
	G15598A	1	0	0	0	0	0	0	0	0	0	0	0	0	1	0
	C15952T	0	0	0	0	0	0	0	0	0	0.11	0	0	0	0	0
	T16176C	0	0	0	0	0	0	0	1	0	0	0	0	0	0	0
	T16242A	0	0	0	0	0	0	0	0	0	0.11	0	0	0	0	0
	C16394T	0	0	0	0	0	0	0	0	0	0	0	0	0	0.03	0
	C16470T	0	0	0	0	0	0	0	0	0	0	0	0	0	0.01	0
	A16856G	0	0	0	0	0	0	0	0	0	0	0	0	0	0.01	0
	C16915T	0	0	1	0	0	0	0	0	0	0	0	0	0	0	0
	C17004T	0	0	1	0	0	0	0	0	0	0	0	0	0	0	0
	T17011A	0	0	0	0	0	0	0	0	0	0	0	0	0	0.01	0
	G17058A	0	1	0	0	0	0	0	0	0	0	0	0	0	0	0
	C17104T	1	0	0	0	0	0	0	0	0	0	0	0	0	1	0
	C17304T	0	0	0	0	0	0	0	0	0	0	0.24	0	0	0	0
	C17375T	0	0	0	0	0	0	0	0	0	0	0	0	0	0.01	0
	G17686T	0	0	0	0	0	0	0	0	0	0	0	0	0	0.03	0
	C17733T	0	0	0	0	1	0	0	0	0	0	0	0	0	0	0
	G18028T	1	0	0	0	0	0	0	0	0	0	0	0	0	1	0
	C18110T	0	0	0	0	0	0	0	0	0	0.11	0	0	0	0	0
	C18176T	0	0	0	0	0	0	0	0	0	0	0	0	0	0	0
	G18255T	0	0	0	0	0	0	0	0	0	0	0	0	0	0.13	0
ORF1b	G18292T	0	0	0	0	0	0	0	0	0	0	0	0	1	0	0
	A18424G	0	0	0	0	0	0	0	0	0	0	0.94	0	0	0	0
	C18440T	0	0	0	0	0	0	0	0	0	0	0	0	0	0.82	0
	T18471C	0	0	0	0	0	0	0	0	0	0	0	0	1	0.14	0
	G18523T	0	0	0	0	0	0	0	0	0	0	0	0	0	0.01	0
	C18555T	0	0	0	0	0	0	0	0	0	0.11	0	0	0	0	0
	T18603C	0	0	0	0	0	0	0	0	0	0	0	0	0	0.01	0
	G18636C	0	0	0	0	0	0	0	0	0	0	0	0	0	0.01	0
	G18703A	0	0	0	0	0	0	0	0	0	0	0.65	0	0	0	0
	C18744T	0	0	0	0	0	1	0	0	0	0	0	0	0	0.01	0

S1 Table. Frequency of SNPs in lineages observed

	C18877T	0	0	0	0	0	0	0	0	0	0	0	0	0	0	1
	C19164T	0	0	0	0	0	0	0	0.1	0	0	0	0	0	0	0
	C19170T	0	0	0	0	0	0	0	0	0	0	0	0	0	0.03	0
	C19263T	0	0	0	0	0	0	0	0	0	0.11	0	0	0	0	0
	C19386A	0	0	0	0	0	0	0	0	0	0	0	0	0	0.01	0
	G19398T	0	0	0	0	0	0	0	0	0	0	0	0	0	0	0
	C19488T	0	0	0	0	0	0	1	0	0	0	0	0	0	0	0
	T19497G	0	0	0	0	0	0	0	0	0	0	0	0	0	0.01	0
	C19610T	0	0	0	0	0	0	0	0	0	0	0	0	0	0.06	0
	A19815T	0	0	0	0	0	0	0	0	0	0	0	0	0	0.03	0
	T19839C	0	0	0	0	0	1	0	0	0	0	0	0	0	0	0
	G19872T	0	0	0	0	0	0	0	0	0	0	0.06	0	0	0	0
	A20268G	1	0	0	0	0	0	0	0.1	0	0	0	0	1	0.99	0
	G20356T	0	0	0	0	0	0	0	0.1	0	0	0	0	0	0	0
	C20451T	1	0	0	0	0	0	0	0.1	0	0	0	0	0	1	0
	A20634R	0	0	0	0	0	0	0	0	0	0	0	0	0	0.01	0
	G20756T	0	0	0	0	0	1	0	0	0	0	0	0	0	0	0
	C20759T	0	0	0	0	0	0	0	0	0	0.11	0	0	0	0	0
	A20785G	0	0	0	0	0	0	0	0	0	0	0	0	0	0.01	0
	A20793T	0	0	0	0	0	0	0	0	0	0	0	0	0	0.01	0
	G21004T	0	0	0	0	0	0	0	0	0	0	0	0	0	0.01	0
	C21058T	0	0	0	0	0	0	0	0	0	0	0	0	0	0.01	0
	T21170G	0	0	0	0	0	0	0	0	0	0	0	0	0	0.01	0
	A21171C	0	0	0	0	0	0	0	0	0	0	0	0	0	0.01	0
	A21194G	0	0	0	0	0	0	0	0.9	0	0	0	0	0	0	0
	G21255C	0	0	0	0	0	0	0	0	0	1	0	0	0	0	0
	C21304T	0	0	0	0	0	0	0	0	0	0	0.94	0	0	0	0
	A21447G	0	0	0	0	0	0	0	0	0	0.22	0	0	0	0	0
	C21549T	0	0	0	0	0	0	0	0	0	0	0	0	0	0.01	0
	C21614T	0	0	0	0	0	0	0	0	0	0.56	0	1	0	0	0
	C21621T	0	0	0	0	0	0	0	0	0	0	0	0	0	0.01	0
	A21631T	0	0	0	0	0	0	0	0	0	0.11	0	0	0	0	0
	T21644C	0	0	0	0	0	0	0	0	0	0	0	0	0	0.01	0
	G21724A	0	0	0	0	0	0	0	0.1	0	0	0	0	0	0	0
	C22000T	0	0	0	0	0	0	0	0	0	0	0	0	0	0.01	0
	G22021T	0	0	0	0	0	0	0	0	0	0	0	0	0	0.01	0
	C22127T	0	0	0	0	0	0	0	0	0	0	0	0	0	0	0
	C22227T	0	0	0	0	0	0	0	0	0	1	0	0	0	0	0
	A22255T	0	0	0	0	0	0	0	0	0	0	0.94	0	0	0	0
	C22444T	0	0	0	0	0	0	0	0	0	0	0	0	0	0	1
	C22879A	1	0	0	0	0	0	0	0	0	0	0	0	0	1	0
	A22974G	0	0	0	0	0	0	0	0	0	0	0.06	0	0	0	0
	A23063T	0	0	0	0	0	0	0	1	0	0	0	0	0	0	0
	A23092G	0	0	0	0	0	0	0	0	0	0	0	0	1	0	0
	C23271A	0	0	0	0	0	0	0	1	0	0	0	0	0	0	0
	A23403G	1	1	1	1	1	1	1	1	1	1	1	1	1	1	1
	C23422T	0	0	0	0	0	1	0	0	0	0	0	0	0	0	0
	C23604G,A	0	1	0	0	0	0	0	1	0	0	0	0	0	0	0
	C23709T	0	0	0	0	0	0	0	1	0	0	0	0	0	0	0
	G23868T	0	0	0	0	0	0	0	0	0	0	0.35	0	0	0	0
Spike	G23876A	0	0	0	0	0	0	0	0	0	0	0	0	0	0	0
	C23939T	0	0	0	0	0	0	0	0	0	0	0	0	0	0.03	0
	G23984A	0	0	0	0	0	0	0	0	0	0	0	0	0	0.01	0
	C24054T	0	0	0	0	0	0	0	0.1	0	0	0	0	0	0	0
	A24067C	0	0	0	0	0	0	0	0	0	0	0.12	0	0	0	0
	G24197T	0	0	0	0	0	0	1	0	0	0	0	0	0	0	0
	G24239T	0	0	0	0	0	0	0	0	0	0	0	0	0	0.03	0
	G24301T	0	0	0	0	0	0	0	0	0	0	0	0	0	0.01	0
	C24334T	0	0	0	0	0	0	0	0	0	0.44	0	0	0	0	0
	C24337G	0	0	0	0	0	0	0	0	0	0	0.06	0	0	0	0
	T24464G	0	0	0	0	0	0	0	0	0	0	0	0	0	0.03	0

S1 Table. Frequency of SNPs in lineages observed

	T24506G	0	0	0	0	0	0	0	1	0	0	0	0	0	0	0
	C24559T	0	0	0	0	0	0	0	0	0.2	0	0	0	0	0	0
	A24591G	0	0	0	0	0	0	0	0	0	0	0.06	0	0	0	0
	A24817C	0	0	0	0	0	0	0	0	0	0	0	0	0	0.03	0
	C24904T	0	0	0	1	0	0	0	0	0	0	0	0	0	0	0
	T24910C	1	0	0	0	0	0	0	0	0	0	0	0	0	1	0
	G24914C	0	0	0	0	0	0	0	1	0	0	0	0	0	0	0
	G25049T	0	0	0	0	0	0	0	0	0	0	0	0	0	0.01	0
	G25088T	0	0	0	0	0	0	0	0	0	0	0	0	0	0.01	0
	T25090C	0	0	0	0	0	0	0	0	0	0	0	0	0	0.01	0
	C25350T	0	0	0	0	0	0	0	0	0	0	0	0	0	0.01	0
	C25380T	0	0	0	0	0	0	0	0	0	0	0	0	0	0.03	0
Orf3a	C25521T	0	0	1	0	0	0	0	0	0	0	0	0	0	0.03	0
	G25563T	0	0	0	0	0	0	0	0	0	0	1	0	0	0	1
	G25606T	0	0	0	0	0	0	0	0	0	0	0.06	0	0	0	0
	C25614T	0	0	0	0	0	0	0	0	0	0.11	0	0	0	0	0
	G25619C	0	0	0	0	0	0	0	0	0	0	0	0	0	0.01	0
	G25699T	0	1	0	0	0	0	0	0	0	0	0	0	0	0	0
	G25720T	0	0	0	0	0	0	0	0	0	0	0.12	0	0	0	0
	G25726T	0	0	0	0	0	0	0	0	0	0	0	1	0	0	0
	A25806T	0	0	0	0	0	0	0	0	0	0	0	0	0.14	0	0
	G25855T	0	0	0	0	0	0	0	0	0	0	0.06	0	0	0	0
	G25907T	0	0	0	0	0	0	0	0	0	0	0.94	0	0	0	0
	G25947T	0	0	0	0	0	0	0	0	0	0	0	0	0	0	0
	G26062T	0	0	1	0	0	0	0	0	0	0	0	0	0	0	0
	T26171A	0	0	0	0	0	0	1	0	0	0	0	0	0	0	0
E	C26313T	0	0	0	0	0	0	0	0	0	0	0	0	0	0.92	0
	T26424C	0	0	0	0	0	0	0	0	0	0.11	0	0	0	0	0
	G26467C	0	0	0	0	0	0	0	0.1	0	0	0	0	0	0.04	0
M	A26702T	0	0	1	0	0	0	0	0	0	0	0	0	0	0	0
	G26730T	0	0	0	0	0	0	0	0	0	0	0	0	0	0.01	0
	C26735T	0	0	0	0	0	0	0	0	0	0	0	0	0	0	1
	C26801G,T	0	0	0	0	0	0	0	0	0	1	0	0	0	0.03	0
	G26834T	0	0	0	0	0	0	0	0	0	0	0	0	0	0.01	0
	C26873T	0	0	0	0	0	0	0	0	0	0	0	0	0	0.01	0
	C26895A	0	1	0	0	0	0	0	0	0	0	0	0	0	0	0
	A26896T	0	0	0	0	0	0	0	0	0	0	0	0	0	0.01	0
	T26972C	1	0	0	0	0	0	0	0.1	0	0	0	0	0	1	0
	C27128T	0	0	0	0	0	0	0	0	0	0	0	0	0	0.04	0
Orf7a	C27476T	0	0	0	0	0	0	0	0	0	0	0	0	0	0.01	0
	C27630T	0	0	0	0	0	0	0	0	0	0.11	0	0	0	0	0
	G27754T	0	0	0	0	0	0	0	0	0	0	0	0	0	0.01	0
Orf7b	C27769T	0	0	0	0	0	0	0	0	0	0.22	0	0	0	0	0
	C27800A	1	0	0	0	0	0	0	0	0	0	0	0	0	0.99	0
	C27804T	0	0	0	0	0	0	0	0	0	0	0.94	0	0	0	0
	C27847A	0	0	0	0	0	0	0	0	0	0	0	0	0	0.03	0
	C27858A	0	0	0	0	0	0	0	0	0	0	0	0	0	0.01	0
Orf8	G27877T	0	0	0	0	0	0	0	0.1	0	0	0	0	0	0	0
	C27944T	0	0	0	0	0	0	0	0	0	1	0	0	0	0.01	0
	C27964T	0	0	0	0	0	0	0	0	0	0	1	0	0	0	0
	C27972T	0	0	0	0	0	0	0	1	0	0	0	0	0	0	0
	C27999A	0	1	0	0	0	0	0	0	0	0	0	0	0	0	0
	C28000T	0	0	0	0	0	0	0	0	0	0	0	0	0	0	1
	G28048T	0	0	0	0	0	0	0	1	0	0	0	0	0	0.03	0
	T28061C	0	1	0	0	0	0	0	0	0	0	0	0	0	0.01	0
	G28077T	0	0	0	0	0	0	1	0	0	0	0	0	0	0	0
	G28079C	0	0	0	0	0	0	0	0	0	0	0	0	0	0.01	0
	C28087T	0	0	1	0	0	0	0	0	0	0	0	0	0	0	0
	A28111G	0	0	0	0	0	0	0	1	0	0	0	0	0	0	0
	G28166A	0	0	0	0	0	0	0	0	0	0	0	0	0	0.06	0

S1 Table. Frequency of SNPs in lineages observed

	G28209T	0	0	0	0	0	0	0	0.1	0	0	0	0	0	0.01	0
	G28237T	0	0	0	0	0	0	0	0	0	0	0	0	0	0.01	0
N	G28280C	0	0	0	0	0	0	0	1	0	0	0	0	0	0	0
	A28281T	0	0	0	0	0	0	0	1	0	0	0	0	0	0	0
	T28282A	0	0	0	0	0	0	0	1	0	0	0	0	0	0	0
	G28321K	0	0	0	0	0	0	0	0	0	0	0.06	0	0	0	0
	G28373T	0	0	0	0	0	0	0	0.1	0	0	0	0	0	0	0
	A28381C	0	0	1	0	0	0	0	0	0	0	0	0	0	0	0
	G28451C	0	0	0	0	0	0	0	0	0	0.11	0	0	0	0	0
	C28472T	0	0	0	0	0	0	0	0	0	0	0.94	0	0	0	0
	C28500T	0	0	0	0	0	0	1	0	0	0	0	0	0	0	0
	C28657T	0	0	0	0	0	0	0	0	0	0.11	0	0	0	0	0
	C28724T	0	0	0	0	0	0	0	0	0	0	0	0	0	0	1
	G28739T	0	0	0	0	0	0	0	0.1	0	0	0	0	0	0	0
	C28849T	0	0	0	0	0	0	0	0	0	0	0.06	0	0	0	0
	C28854T	0	0	0	0	0	0	0	0	0	0	0	0	1	0	1
	C28869T	0	0	0	0	0	0	0	0	0	0	0.94	0	0	0	0
	G28881A	0	1	1	1	1	1	1	1	1	0	0	0	0	0	0
	G28882A	0	1	1	1	1	1	1	1	1	0	0	0	0	0	0
	G28883C	0	1	1	1	1	1	1	1	1	0	0	0	0	0	0
	C28887T	0	0	0	0	0	0	0	0	0	0	0	0	0	0.01	0
	C28932T	0	0	0	0	0	0	0	0	0	1	0	0	0	0	0
	G28975T	0	0	0	0	0	0	0	0	0	0	0	0	0	0	0
	C28977T	0	0	0	0	0	0	0	0.9	0	0	0	0	0	0	0
	C29095T	0	0	0	0	1	0	0	0	0	0	0	0	0	0	0
	C29200T	0	0	0	0	0	0	0	0	0	0	0	0	0	0.03	0
	C29218T	0	0	0	0	0	0	0	0	0	0	0	0	0	0.01	0
	G29254A,T	0	0	0	0	0	0	1	0	0	0	0.06	0	0	0.03	0
	G29266A	0	0	0	0	0	0	0	0	0	0	0	0	0	0.01	0
	C29296T	0	0	0	0	0	0	0	0	0	0.11	0	0	0	0	0
	G29384T	0	0	0	0	0	0	0	0	0	0	0	0	0	0.01	0
	G29405C	0	0	0	0	0	1	0	0	0	0	0	0	0	0	0
	G29468T	0	0	0	0	0	0	0	0.1	0	0	0	0	0	0.01	0
	G29474T	0	0	0	0	0	0	1	0	0	0	0.06	0	0	0.01	0
	G29527A	0	0	0	0	0	0	0	0	0	0	0	0	0.29	0	0
Orf10	G29645T	0	0	0	0	0	0	0	0	0	1	0	0	0	0.01	0
	G29648T	0	0	0	0	0	0	0	0	0	0	0	0	0	0.01	0
3'UTR	G29692T	0	0	0	0	0	1	0	0	0	0	0.06	0	0	0	0
	G29734T,C	1	0.5	0	0	0	0	0	0.1	0	0	0	0	0	1	0
	G29751C,A	0	0	0	0	0	0	1	0	0	0	0	0	0	0.03	0
	G29755T	0	0	0	0	0	0	0	0	0	0.22	0	0	0	0	0
	G29764T	0	0	0	0	0	0	0	0	0	0	0.06	0	0	0	0
	G29766T	0	0	0	0	0	0	0	0	0	0	0.06	0	0	0	0
	C29774T	0	0	0	0	0	0	0	0	0	0	0	0	0	0.01	0
	C29835A	0	0	0	0	0	0	0	0	0	0.11	0	0	0	0	0
	G29868T	0	0	0	0	0	0	0	0	0	0	0.06	0	0	0	0

Single nucleotide polymorphisms (SNPs) were identified in the genomes with respect to the reference sequence (NC_045512). The frequency of SNPs is calculated by the number of sequences with the SNP/total number of sequences per lineage. SNPs are ordered by their position on the genome. Number of sequences of each lineage are indicated in brackets in the header. SNPs with frequency >75% are marked in green, SNPs with frequency between 50% and 75% are marked in yellow.
